# Detecting beats in the photoplethysmogram: benchmarking open-source algorithms

**DOI:** 10.1088/1361-6579/ac826d

**Published:** 2022-08-19

**Authors:** Peter H Charlton, Kevin Kotzen, Elisa Mejía-Mejía, Philip J Aston, Karthik Budidha, Jonathan Mant, Callum Pettit, Joachim A Behar, Panicos A Kyriacou

**Affiliations:** 1 Department of Public Health and Primary Care, University of Cambridge, Cambridge, CB1 8RN, United Kingdom; 2 Research Centre for Biomedical Engineering, City, University of London, London, EC1V 0HB, United Kingdom; 3 Faculty of Biomedical Engineering, Technion-IIT, Israel; 4 Department of Mathematics, University of Surrey, Guildford, Surrey GU2 7XH, United Kingdom

**Keywords:** atrial fibrillation, beat detection, electrocardiogram, heartbeat, photoplethysmography, pulse wave

## Abstract

The photoplethysmogram (PPG) signal is widely used in pulse oximeters and smartwatches. A fundamental step in analysing the PPG is the detection of heartbeats. Several PPG beat detection algorithms have been proposed, although it is not clear which performs best. *Objective:* This study aimed to: (i) develop a framework with which to design and test PPG beat detectors; (ii) assess the performance of PPG beat detectors in different use cases; and (iii) investigate how their performance is affected by patient demographics and physiology. *Approach:* Fifteen beat detectors were assessed against electrocardiogram-derived heartbeats using data from eight datasets. Performance was assessed using the *F*_1_ score, which combines sensitivity and positive predictive value. *Main results:* Eight beat detectors performed well in the absence of movement with *F*_1_ scores of ≥90% on hospital data and wearable data collected at rest. Their performance was poorer during exercise with *F*_1_ scores of 55%–91%; poorer in neonates than adults with *F*_1_ scores of 84%–96% in neonates compared to 98%–99% in adults; and poorer in atrial fibrillation (AF) with *F*_1_ scores of 92%–97% in AF compared to 99%–100% in normal sinus rhythm. *Significance:* Two PPG beat detectors denoted ‘MSPTD’ and ‘qppg’ performed best, with complementary performance characteristics. This evidence can be used to inform the choice of PPG beat detector algorithm. The algorithms, datasets, and assessment framework are freely available.

## 1. Introduction

The photoplethysmogram (PPG) signal is acquired by a range of clinical and consumer devices, from pulse oximeters to smartwatches (Allen 2007, Charlton and Marozas 2022). It exhibits a pulse wave for each heartbeat, caused by the ejection of blood from the heart into the circulation. A wealth of physiological information can be deduced from the timing and shape of PPG pulse waves (Charlton *et al* 2022). Consequently, a fundamental step in analysing the PPG is to detect individual pulse waves, corresponding to individual heartbeats. Indeed, several beat detection algorithms have been developed for the PPG, although it is not yet known how their performance compares.

It is important to assess the performance of beat detectors in different use cases where PPG signals can have different morphologies and levels of artifact (Charlton *et al* 2022). Specifically, pulse oximeters acquire PPG signals at the finger close to major arteries, often with little motion artifact. In contrast, smart wearables such as smartwatches and fitness bands acquire the PPG at the wrist further from major arteries, often in challenging conditions such as during exercise. Assessing the performance of beat detectors across different use cases would allow one to select the best beat detector for a particular use case, and to understand its expected performance.

It is also important to investigate the impact of patient demographics and physiology on performance. First, it is important to assess performance during arrhythmias, since the PPG is now being used to identify atrial fibrillation (AF) (Perez *et al* 2019). Second, performance should be compared between ethnicities, as the performance of pulse oximeters has been found to be related to ethnicity (Sjoding *et al* 2020). Third, it is important to assess whether performance differs in babies, who have higher heart rates (HRs) than adults (Fleming *et al* 2011). Assessing the impact of patient demographics and physiology on performance could highlight areas for future algorithm development.

This study aimed to: (i) develop an assessment framework with which to design and test PPG beat detectors; (ii) assess the performance of several beat detectors in different use cases; and (iii) investigate how their performance is affected by patient demographics and physiology. Fifteen open-source beat detectors were assessed against reference beats from electrocardiogram (ECG) signals in eight freely available datasets. This study builds on previous work which assessed the performance of four beat detectors on a single dataset (Kotzen *et al* 2021), whereas this study assessed fifteen beat detectors across eight datasets.

## 2. Materials and methods

Ethical approval was not required for this study as it used pre-existing, anonymised data.

### 2.1. Datasets

The datasets used in this study are summarised in table 1, and are now described.

**Table 1.** Datasets used to assess the performance of PPG beat detectors.

Dataset	Subjects	PPG equipment	Reference beats	Duration (mins):	Total beats
				med (quartiles)	
*Hospital monitoring (high-quality data)*					

CapnoBase	42 patients undergoing elective surgery and routine anaesthesia (Karlen *et al* 2013).	Pulse oximeter at 300 Hz (upsampled from 100 Hz during acquisition)	Manual annotations of ECG (300 Hz)	7.7 (7.0–7.8)	24,945
BIDMC	53 critically-ill adult patients, a subset of the MIMIC II dataset (Pimentel *et al* 2017).	Bedside monitor at 125Hz (mostly finger PPG recordings)	ECG-derived QRS detections (125 Hz)	7.4 (6.9-7.7)	32,484

*Hospital monitoring (real-world data)*					

MIMIC PERform Training Dataset	200 critically-ill patients during routine clinical care (100 adults, 100 neonates).	Bedside monitor at 125 Hz (mostly finger PPG recordings)	ECG-derived QRS detections (125 Hz)	5.7 (3.6-7.8)	115,941
MIMIC PERform Testing Dataset	200 critically-ill patients during routine clinical care (100 adults, 100 neonates).	Bedside monitor at 125 Hz (mostly finger PPG recordings)	ECG-derived QRS detections (125 Hz)	All: 5.2 (3.4-7.9); Adults: 7.7 (5.1-8.7); Neonates: 4.0 (2.6-5.3)	All: 116,585; Adults: 57,013; Neonates: 59,572
MIMIC PERform AF Dataset	35 critically-ill adults during routine clinical care (19 in AF, 16 not in AF), using AF labels provided by cardiologists (Bashar *et al* 2019, Bashar 2020).	Bedside monitor at 125Hz (mostly finger PPG recordings)	ECG-derived QRS detections (125 Hz)	AF: 17.8 (15.2-19.6); non-AF: 18.6 (17.3-19.4)	AF: 29,592; non-AF: 22,477
MIMIC PERform Ethnicity Dataset	200 critically-ill adults during routine clinical care (100 of Black ethnicity, 100 of White).	Bedside monitor at 125 Hz (mostly finger PPG recordings)	ECG-derived QRS detections (125 Hz)	Black: 8.0 (5.6-9.3); White: 7.0 (3.4-8.8)	Black: 61,756; White: 51,230

*Wearable data during different emotions*					

WESAD	15 subjects during a laboratory-based protocol designed to induce different emotions (Schmidt *et al* 2018).	Wristband (Empatica E4) at 64Hz.	ECG-derived QRS detections (700 Hz)	Baseline: 19.1 (18.9-19.3); Amusement: 5.8 (5.8-5.8); Meditation: 6.3 (6.1-6.3); Stress: 10.3 (10.1-10.8)	Baseline: 20,519; Amusement: 6,213; Meditation: 6,395; Stress: 15,282

*Wearable data during activities of daily living*					

PPG-DaLiA	15 subjects during a protocol of activities of daily living (Reiss *et al* 2019).	Wristband (Empatica E4) at 64Hz.	Manual annotations of ECG (700 Hz)	Sitting: 9.8 (9.7–0.0); Working: 19.9 (19.7–20.5); Cycling: 7.8 (6.7–8.2); Walking: 10.8 (9.5–11.5); Lunch break: 32.4 (28.7–37.2); Car driving: 15.0 (14.1–15.8); Stair climbing: 7.5 (6.8–7.7); Table soccer: 4.8 (4.5–5.2)	Sitting: 9,022; Working: 21,272; Cycling: 13,956; Walking: 15,062; Lunch break: 37,247; Car driving: 18,883; Stair climbing: 12,466; Table soccer: 6,625

For each dataset, the table indicates the duration of recordings and the total number of beats used in the analysis (shown for the MPSTD beat detector).

#### 2.1.1. Hospital monitoring

A total of six datasets were used to assess performance during hospital monitoring: the CapnoBase and BIDMC datasets (which contain high-quality data), and four novel datasets extracted from the MIMIC Database (which contain real-world data).

The CapnoBase and BIDMC datasets were originally designed for developing and assessing PPG signal processing algorithms. They contain high-quality ECG and PPG signals with little artifact. Therefore, the performance of beat detectors on these datasets represents the best possible performance that could be expected in hospital monitoring. CapnoBase (Karlen *et al* 2013) contains data from 42 paediatric and adult subjects undergoing elective surgery and anaesthesia. BIDMC (Pimentel *et al* 2017) contains data from 53 adults receiving critical care on a Medical Intensive Care Unit (46 subjects), Coronary Care Unit (6), or Surgical Intensive Care Unit (1). The BIDMC dataset was originally derived from the MIMIC-II Database (Goldberger *et al* 2000, Saeed *et al* 2011).

In addition, four novel datasets were extracted from the MIMIC-III Database (Goldberger *et al* 2000, Johnson *et al* 2016) for this study. These are named the ‘MIMIC PERform’ Datasets, as they contain (P) PPG, (E) ECG and (R) Respiration signals. These datasets were designed to be representative of real-world critical care data: their signals contain motion artifact and some low-quality periods. The MIMIC PERform Training and Testing Datasets each contain data 10 minutes of data from 200 patients, consisting of 100 adults and 100 neonates. The MIMIC PERform Testing Dataset was used to compare performance between adults and neonates in this study. The MIMIC PERform AF Dataset contains 20 minutes of data from 19 patients in AF, and 16 patients in normal sinus rhythm (non-AF). It was used to compare performance between AF and normal sinus rhythm. Labels of AF were obtained from manual annotations by cardiologists (Bashar *et al* 2019, Bashar 2020). The MIMIC PERform Ethnicity Dataset contains 10 minutes of data from 100 Black and 100 White subjects. It was used to compare performance between Black and White subjects, in keeping with (Sjoding *et al* 2020). All MIMIC PERform Datasets were extracted from the MIMIC-III Waveform Database, except for the Ethnicity Dataset, which was extracted from the MIMIC-III Matched Waveform Database (Moody *et al* 2020). Data were extracted by searching for MIMIC records which met the following criteria: (i) contain the required signals (PPG, ECG, and for all except the AF Dataset, respiration); (ii) are of sufficient duration (≥10 minutes in the case of the Training, Testing and Ethnicity Datasets, and ≥20 minutes in the case of the AF Dataset); and (iii) contain minimal flat line segments (indicating sensor disconnection or saturation). The MIMIC Perform Datasets are available in (Charlton 2022b).

#### 2.1.2. Wearable data

Two wearable datasets were used, each containing wrist PPG signals acquired using a wearable Empatica E4 device. The WESAD dataset was acquired during a protocol designed to induce different emotions: baseline, meditation, amusement, and stress. It contains data from 15 subjects, including 3 females, with a median age (lower—upper quartiles) of 27 (26–28) years, and BMI of 23 (22–25) kgm^−2^. The PPG-DaLiA dataset was acquired during a protocol of activities of daily living, including: sitting, working, cycling, and running. It contains data from 15 subjects, including 3 females aged 28 (24–36) years, with a BMI of 22 (21–23) kgm^−2^, and skin types on the Fitzpatrick scale of: 2 (1 subject), 3 (11 subjects), and 4 (3 subjects).

### 2.2. PPG beat detection

**Figure 1.**
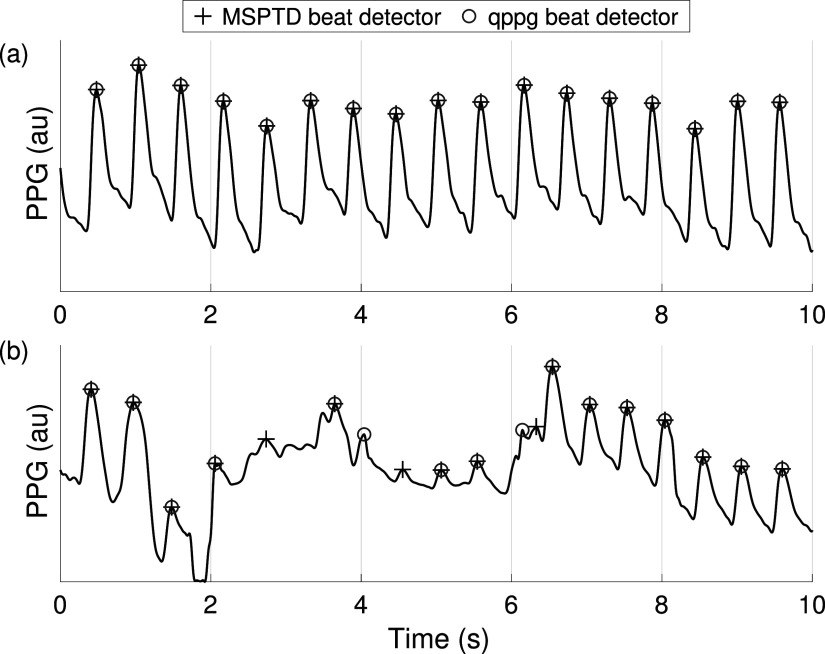
Detecting beats in the photoplethysmogram (PPG): PPG pulse peaks detected by two beat detectors. (a) shows a high quality segment in which beats were accurately detected by both beat detectors; (b) includes a period of low quality between 1 and 7 s in which the two beat detectors disagreed. *au—arbitrary units*.

First, any PPG signals sampled at over 100 Hz were resampled at this frequency to reduce the time for computational analysis. For signals sampled at multiples of 100 Hz, this was performed using downsampling, and for other signals it was performed using resampling with an antialiasing lowpass filter. Second, signals were band-pass filtered between 0.67 and 8.0 Hz to eliminate non-cardiac frequencies. Third, beats were detected using fifteen open-source PPG beat detectors in turn, as demonstrated for two beat detectors in figure 1. The beat detectors are described in table 2. Beat detection was performed on 20 s windows of PPG signal, overlapping by 5 s. Repeated beat detections due to overlapping windows were eliminated. This approach ensured that beat detectors were not penalised for missing beats at the start or end of a window. Fourth, windows were excluded if they contained a flat line lasting more than 0.2 s (typically caused by sensor disconnection or signal ‘clipping’). The beat detectors are available in (Charlton 2022a).

**Table 2.** PPG Beat Detectors.

Beat Detector	Implementing Author	Original Author	Description
**ABD**: Automatic Beat Detection (Aboy *et al* 2005)	P. Charlton	M. Aboy *et al*	The PPG is strongly filtered to retain frequencies around an initial heart rate estimate, differentiated, and peaks are detected above the 75th percentile. Beats are identified as peaks in a weakly filtered PPG immediately following each peak identified in the differentiated signal.
**AMPD**: Automatic Multiscale Peak Detection (Scholkmann *et al* 2012)	P. Charlton	F. Scholkmann *et al*	The PPG is detrended and segmented into 6s windows. A local maxima scalogram (LMS) is calculated: a matrix of random numbers, where the rows correspond to different scales (ranging from one sample to half the window duration), and the columns indicate PPG samples. The LMS values are set to zero when a PPG sample is higher than its neighbours at that particular scale. The LMS is truncated to only include scales smaller than the scale at which the most local maxima were identified. Beats are identified as samples which are deemed to be local maxima at all remaining scales.
**ATM**: Adaptive Threshold Method (Shin *et al* 2009, Han *et al* 2022)	D. Han	H. Shin *et al*	The PPG is bandpass filtered between 0.5 and 20 Hz. Troughs are identified as local minima which are below an adaptive threshold. The adaptive threshold increases from the value of the previous trough, at a rate related to the PPG amplitude. Any troughs occuring within a period of 0.6 times the previous inter-beat-interval are excluded. The ‘Vmin’ implementation of this beat detector was used, as it performed slightly better than the ‘Vmax’ implementation in initial testing.
**COppg**: Percentile Peak Detector (Orphanidou *et al* 2015)	P. Charlton, C. Orphanidou, A. Darrell	C. Orphanidou *et al*	In each 10 s PPG segment, beats are identified as peaks which are sufficiently close to (or above) the 90th percentile of the PPG signal, using adaptive filtering.
**ERMA**: Event-Related Moving Averages (Elgendi *et al* 2013)	E. Mejía-Mejía	M. Elgendi *et al*	The PPG is bandpass filtered between 0.5 and 8Hz, rectified to eliminate values below zero, and squared. Two moving averages are calculated: (i) MA_peak_, a moving average of period 111 ms, emphasising systolic peaks; and (ii) MA_beat_, a moving average of period 667 ms, emphasising individual beats. Beats are identified as maxima within periods lasting ≥111 ms where MA_peak_ *>*MA_beat_ + *α* (where *α* is a threshold).
**HeartPy**(van Gent *et al* 2019, 2019)	P. Charlton	P. van Gent *et al*	The PPG is squared and normalised. Peaks are detected as maxima above a moving average (of period 0.75s). This is repeated for moving averages of different amplitudes, producing a set of peaks for each amplitude. The set of peaks which produces a plausible HR and the lowest variability in inter-beat intervals (IBIs) is selected as the set of beats. Beats which result in outlying IBIs are eliminated.
**IMS:** Incremental Merge Segmentation (Karlen *et al* 2012)	M. Pimentel	W. Karlen *et al*	Beats are detected at the end of continuous positive gradient segments (systolic upslopes) with an acceptable amplitude and duration, where the amplitude thresholds are adaptively calculated.
**MSPTD**: Multi-Scale Peak & Trough Detection (Bishop and Ercole 2018)	S. Bishop	S. Bishop & A. Ercole	A modification of AMPD in which LMS matrices are calculated for both local maxima and local minima, so the algorithm detects both peaks and onsets. MSPTD also contains some optimisations to improve computational efficiency.
**PDA**: Peak Detection algorithm (Argüello Prada and Serna Maldonado 2018)	E. Mejía-Mejía	E.J. Argüello Prada & R.D. Serna Maldonado	Systolic peaks are identified as peaks which follow an upslope (i.e., period of positive gradient) lasting ≥60% of the duration of the upslope leading to the previously detected systolic peak.
**PWD**: Pulse Wave Delineator (Li *et al* 2010)	B.N. Li	B.N. Li *et al*	Pulse onsets and pulse peaks are identified from zero-crossing points in the first derivative of the PPG: onsets are identified as zero-crossing points before a maximal deflection, and peaks are identified as zero-crossing points immediately following maximal deflections.
**Pulses**: PPG Pulses Detector (Lázaro *et al* 2014)	J. Lazaro, M. Llamedo Soria	J. Lazaro *et al*	Peaks are identified in the differentiated PPG using an adaptive filter set to the amplitude of the previous peak, and decreases for a period after that peak at a rate dependent on previous inter-beat intervals. Beats are identified as maxima in the PPG within 300ms of each peak in the differentiated PPG.
**qppg**: Adapted Onset Detector (Vest *et al* 2018)	W. Zong, G. Moody, Q. Li	W. Zong	Systolic upslopes are detected from a signal generated with a slope sum function, which sums the magnitudes of the PPG upslopes in the previous 0.17 s. Adaptive thresholding is used to identify systolic upslopes in this signal. The ’qppgfast’ implementation of this beat detector was used, after testing showed it performed similarly to the original ’qppg’ implementation.
**SPAR**: Symmetric Projection Attractor Reconstruction (Pettit and Aston, [Bibr pmeaac826dbib34])	C. Pettit & P.J. Aston	C. Pettit *et al*	The PPG is segmented into 20 s windows and time delay coordinates are used to represent it in 7-dimensional phase space with the time delay set to one seventh of the average inter-beat interval. The Symmetric Projection Attractor Reconstruction method is then used to construct an appropriate 2-dimensional projection of the phase space (Aston *et al* 2018, Lyle and Aston 2021). Beats are identified as times at which the orbit crosses the *x*-axis. This implementation uses information from previous windows to inform beat detections in the current window.
**SWT**: Stationary Wavelet Transform (Vadrevu and Sabarimalai Manikandan 2019)	D. Han	S. Vadrevu & M. Sabarimalai Manikandan	The PPG is decomposed using the Stationary Wavelet Transform. Multi-scale sum and products of selected detail subbands are calculated to emphasise systolic upslopes. An envelope is then extracted by: adaptive thresholding to reduce the influence of noise; calculating the Shannon entropy; and smoothing the result. Finally, beats are identified in the envelope using a Gaussian derivative filter.
**WFD**: Wavelet Foot Delineation (Conn and Borkholder 2013)	E. Mejía-Mejía	N. Conn & D. Borkholder	The PPG is bandpass filtered between 0.5 and 8 Hz, and interpolated to 250 Hz. It is decomposed using a wavelet transform, retaining the fifth wavelet scale for analysis. This signal is rectified and squared to eliminate values below zero. Regions containing beats are identified as those where the signal exceeds a low-pass filtered version of the signal. The timing of the beat within each region is identified as the first zero-crossing of the third derivative, or failing that, the maximum in the second derivative.

For consistency, each beat detector’s annotations were used to obtain the corresponding middle-amplitude point of the systolic upslope on each detected PPG pulse wave (Peralta *et al* 2019), which was used for analysis. This point has been found to provide more accurate timings than peaks or onsets (Peralta *et al* 2019).

### 2.3. Reference ECG beat detection

The CapnoBase and PPG-DaLiA datasets contain manual beat annotations which were used as reference beats. In the remaining datasets reference beats were obtained from simultaneous ECG signals by: (i) detecting beats using two separate ECG beat detectors; (ii) identifying ‘correct’ beats as those which both beat detectors detected within 150 ms of each other; and (iii) excluding from the analysis any 20 s windows in which the two beat detectors did not agree. The two beat detectors were: the ‘jqrs’ ECG beat detector, which is based on the Pan and Tompkins method (Behar *et al* 2014, Johnson *et al* 2014) and the ‘rpeakdetect’ ECG beat detector (Clifford[Bibr pmeaac826dbib14]).

### 2.4. Aligning PPG beats with reference ECG Beats

**Figure 2.**
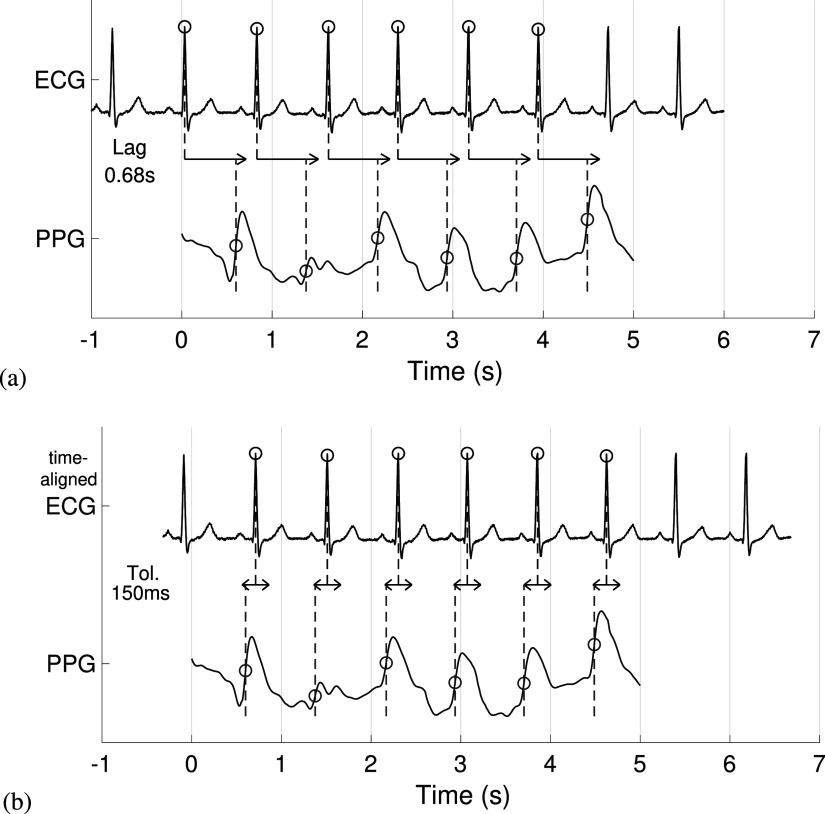
**Comparing PPG-derived beats with reference beats:** (a) Time-alignment of electrocardiogram (ECG) and photoplethysmogram (PPG) signals. The time lag between ECG and PPG signals (0.68 s in this case) was automatically identified from ECG and PPG beat timings. (b) Assessing the ability of a beat detector to detect beats in the PPG. Those beats detected in the PPG (red circles) which occured within ± 150ms of time-aligned reference ECG beats were deemed to be correct.

PPG and ECG signals were not necessarily precisely aligned, so the timings of PPG-derived beats and reference ECG-derived beats were aligned as follows. The time difference between each ECG-derived beat and its closest PPG-derived beat was calculated. Those ECG-derived beats for which the absolute time difference was <150 ms were determined to be correctly identified. This process was repeated when offsetting the beats by lags of −10 to 10 s, in increments of 20 ms. The lag which resulted in the highest proportion of beats being correctly identified was accepted as the true lag and used to synchronise the timings of beats. Figure 2(a) shows an example of this time-alignment.

### 2.5. Statistical analysis

The ability of beat detectors to detect beats was assessed by comparing PPG-derived beats with reference beats. Reference beats were determined to be correctly identified if the closest PPG-derived beat was within ±150 ms of a reference beat, as shown in figure 2(b). For each recording, the numbers of reference beats (*n*_ref_), PPG-derived beats (*n*_PPG_), and correctly identified beats (*n*_correct_) were used to calculate the following:

\begin{eqnarray*}{\mathrm{s}}{ensitivity}( \% ),\quad \mathrm{Se}=\displaystyle \frac{{n}_{\mathrm{correct}}}{{n}_{\mathrm{ref}}}\times 100\end{eqnarray*}

\begin{eqnarray*}{\mathrm{p}}{ositive}\,{predictive}\,{value}( \% ),\quad \mathrm{PPV}=\displaystyle \frac{{n}_{\mathrm{correct}}}{{n}_{\mathrm{PPG}}}\times 100\end{eqnarray*}

\begin{eqnarray*}{F}_{1}\,\mathrm{Score}( \% ),\quad {{\mathrm{F}}}_{1}=\displaystyle \frac{2\times \mathrm{PPV}\times \mathrm{Se}}{\mathrm{PPV}+\mathrm{Se}}\times 100\end{eqnarray*}

Beat detectors were ranked according to the *F*_1_ score, which is the harmonic mean of sensitivity and PPV.

The accuracy of PPG-derived heart rates (HRs) was assessed by comparing PPG-derived HRs to reference ECG-derived HRs. A HR (in beats per minute, bpm) was calculated at the time of each PPG-derived beat, from the number of PPG-derived beats in the preceding 8 s window (*n*_*beats*_), as

\begin{eqnarray*}{HR}=60\times \displaystyle \frac{{n}_{\mathrm{beats}}-1}{t({n}_{\mathrm{beats}})-t(1)}\end{eqnarray*}

where *t* denotes the times of PPG-derived beats. Each HR signal was interpolated using sample-and-hold interpolation at 50 Hz. Performance was assessed as the mean absolute percentage error (MAPE) between time series. A median MAPE of <10% was deemed to be acceptable for HR monitoring. This was based on the acceptable limits of ±10% stated in the AAMI standard (ANSI/AAMI 2002) and implemented using the MAPE statistic in (Consumer Technology Association 2018), although we note that the true threshold of acceptability is likely to vary between applications (Mühlen *et al* 2021).

Performance statistics are reported as median (25th–75th percentiles). The Wilcoxon rank sum test was used to compare performances between groups, at a significance level of *α* = 0.05. A Holm-Sidak correction was made to correct for multiple comparisons.

## 3. Results

The main results are summarised in table 3. This table reports the performance of beat detectors (*F*_1_ score) and their performance for HR monitoring (HR MAPE). Results are provided for the best-performing beat detectors (found to be MSPTD and qppg, as detailed in section 3.2), and all beat detectors (reported as the range in performance metrics from the worst to the best performance).

**Table 3.** The performance of beat detectors in different use cases.

Dataset	median *F*_1_ score (%)			median HR MAPE (%)		
	MSPTD	qppg	All (min—max)	MSPTD	qppg	All (min—max)
*Hospital Monitoring (high-quality data)*						

CapnoBase	99.9	99.9	97.1-99.9	0.2	0.2	0.2-3.7
BIDMC	99.7	99.6	93.4-99.7	0.5	0.7	0.5-6.5

*Hospital Monitoring (real-world data)*						

MIMIC PERform Training Dataset	97.2	96.5	59.0-97.2	2.1	3.7	2.1-49.1
MIMIC PERform Testing Dataset	97.5	96.9	59.0-97.5	2.4	3.5	2.4-51.0
MIMIC PERform Testing Dataset (adults)	98.5	98.0	91.9-98.5	1.1	2.2	1.1-13.5
MIMIC PERform Testing Dataset (neonates)	95.9	95.2	50.7-95.9	4.9	5.5	4.8-59.7
MIMIC PERform AF Dataset (AF)	96.7	97.1	75.3-97.1	4.3	3.3	3.3-34.9
MIMIC PERform AF Dataset (non-AF)	99.7	99.6	91.3-99.7	0.4	0.6	0.4-6.9
MIMIC PERform Ethnicity Dataset (Black)	98.5	98.2	91.2-98.5	1.4	2.3	1.4-9.9
MIMIC PERform Ethnicity Dataset (White)	97.5	97.3	86.6-97.5	2.1	3.5	2.1-14.6

*Wearable data during different emotions*						

WESAD (meditation)	98.2	98.3	71.5-98.3	0.6	1.5	0.6-27.8
WESAD (amusement)	95.6	92.8	43.6-95.6	2.0	4.4	2.0-44.8
WESAD (baseline)	80.1	74.2	37.0-80.1	3.8	8.6	3.8-41.8
WESAD (stress)	70.1	68.7	17.9-70.1	13.2	15.5	13.2-67.7

*Wearable data during activities of daily living*						

PPG-DaLiA (sitting)	95.1	95.1	63.1-95.5	2.5	4.1	2.5-29.9
PPG-DaLiA (working)	81.2	80.0	40.3-81.4	4.3	8.0	4.3-48.6
PPG-DaLiA (cycling)	87.1	90.6	33.6-90.6	13.0	7.0	7.0-69.0
PPG-DaLiA (walking)	72.1	76.9	31.2-76.9	19.1	13.7	13.7-63.2
PPG-DaLiA (lunch break)	66.0	66.8	22.8-66.8	6.7	8.2	6.7-59.7
PPG-DaLiA (car driving)	83.1	80.2	30.5-83.1	5.7	7.8	5.7-61.0
PPG-DaLiA (stair climbing)	71.3	71.9	27.9-71.9	20.1	15.1	15.1-71.9
PPG-DaLiA (table soccer)	65.3	61.0	19.8-65.3	13.9	19.1	13.3-65.7

### 3.1. Performance of beat detectors in different use cases

**Figure 3.**
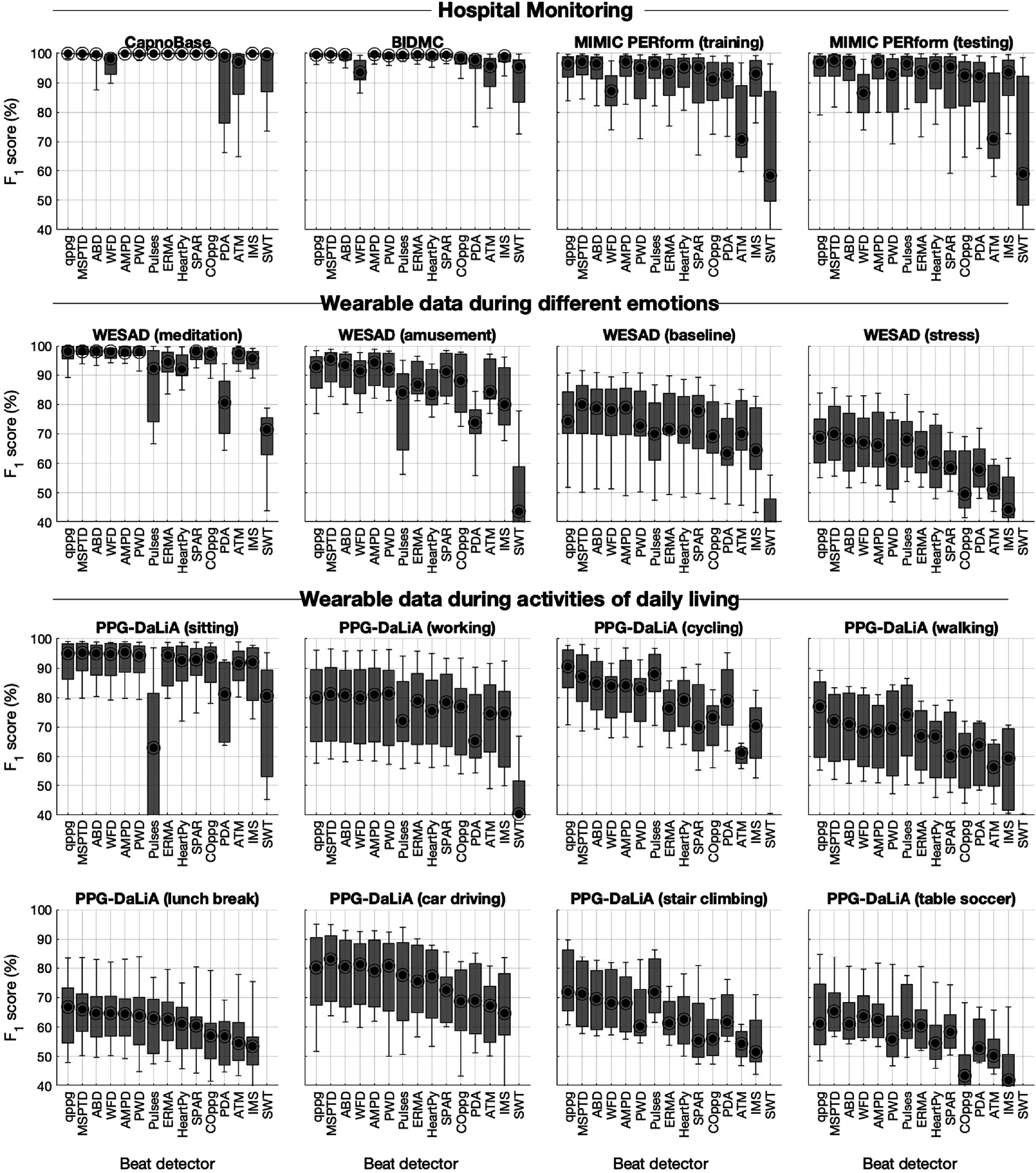
Box plots showing the performance of beat detectors, expressed as the *F*_1_ score. Each graph shows the results for each of the beat detectors on a particular dataset. Performance is shown as the median (circles), inter-quartile range (boxes), and 10th and 90th percentiles (whiskers) across subjects. See table 2 for definitions of beat detectors.

**Figure 4.**
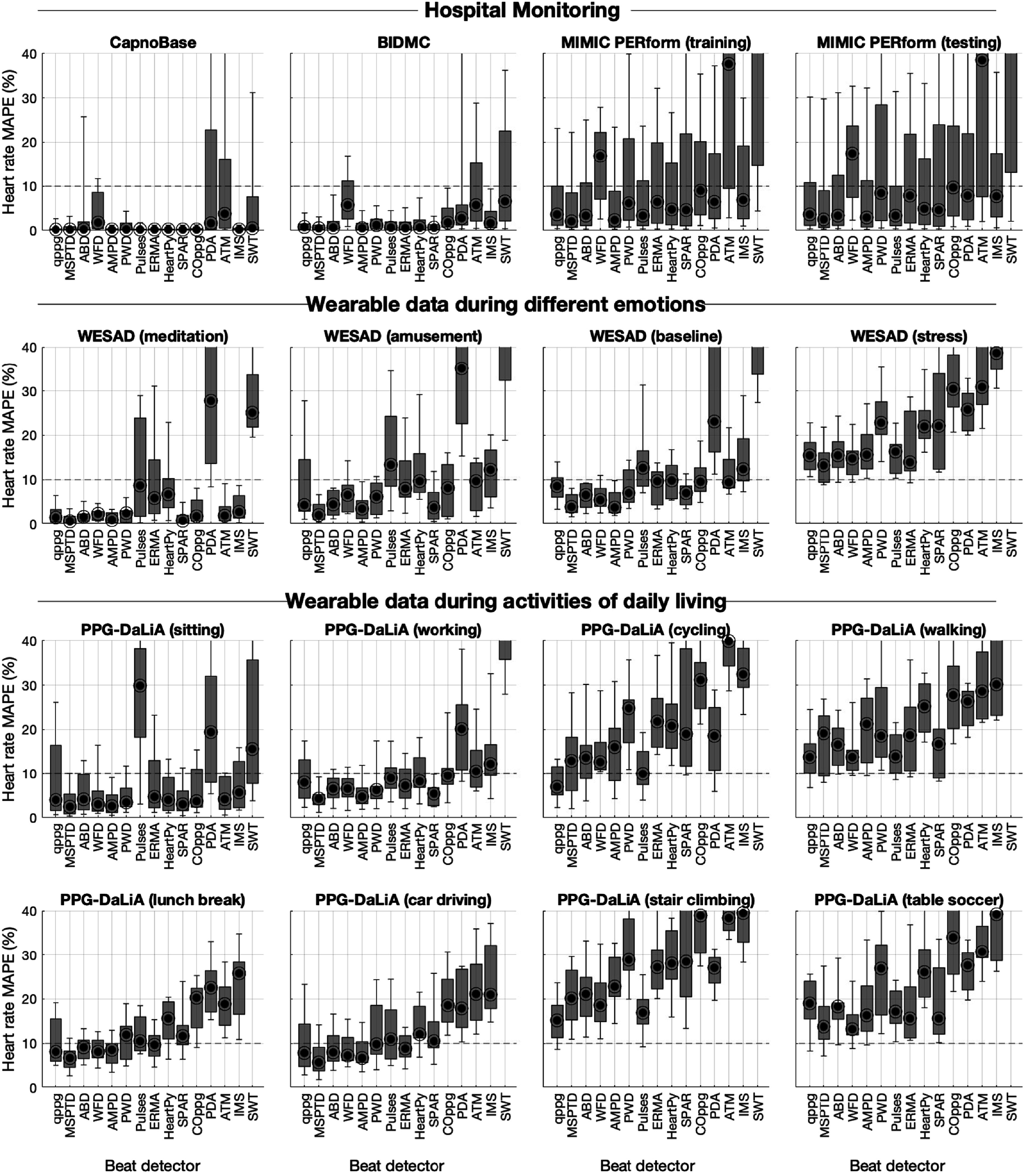
Box plots showing the performance of beat detectors, expressed as the heart rate mean absolute percentage error (MAPE). Each graph shows the results for each of the beat detectors on a particular dataset. Performance is shown as the median (circles), inter-quartile range (boxes), and 10th and 90th percentiles (whiskers) across subjects. Dashed red lines indicate the acceptable performance of 10% MAPE. See table 2 for definitions of beat detectors.

**Figure A1.**
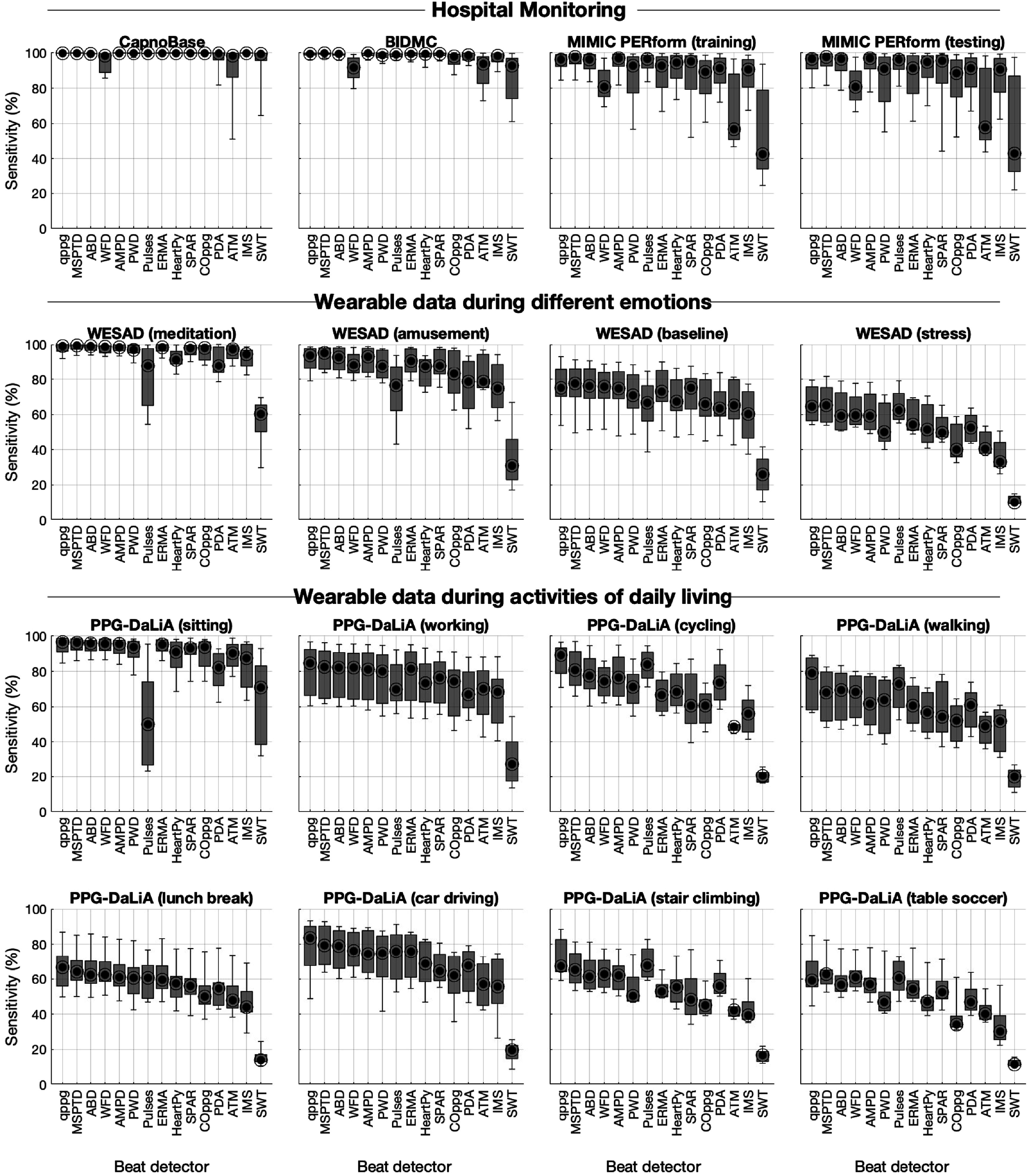
Box plots showing the performance of beat detectors, expressed as the sensitivity. Each graph shows the results for each of the beat detectors on a particular dataset. Performance is shown as the median (circles), inter-quartile range (boxes), and 10th and 90th percentiles (whiskers) across subjects. See table 2 in the main text for definitions of beat detectors.

**Figure A2.**
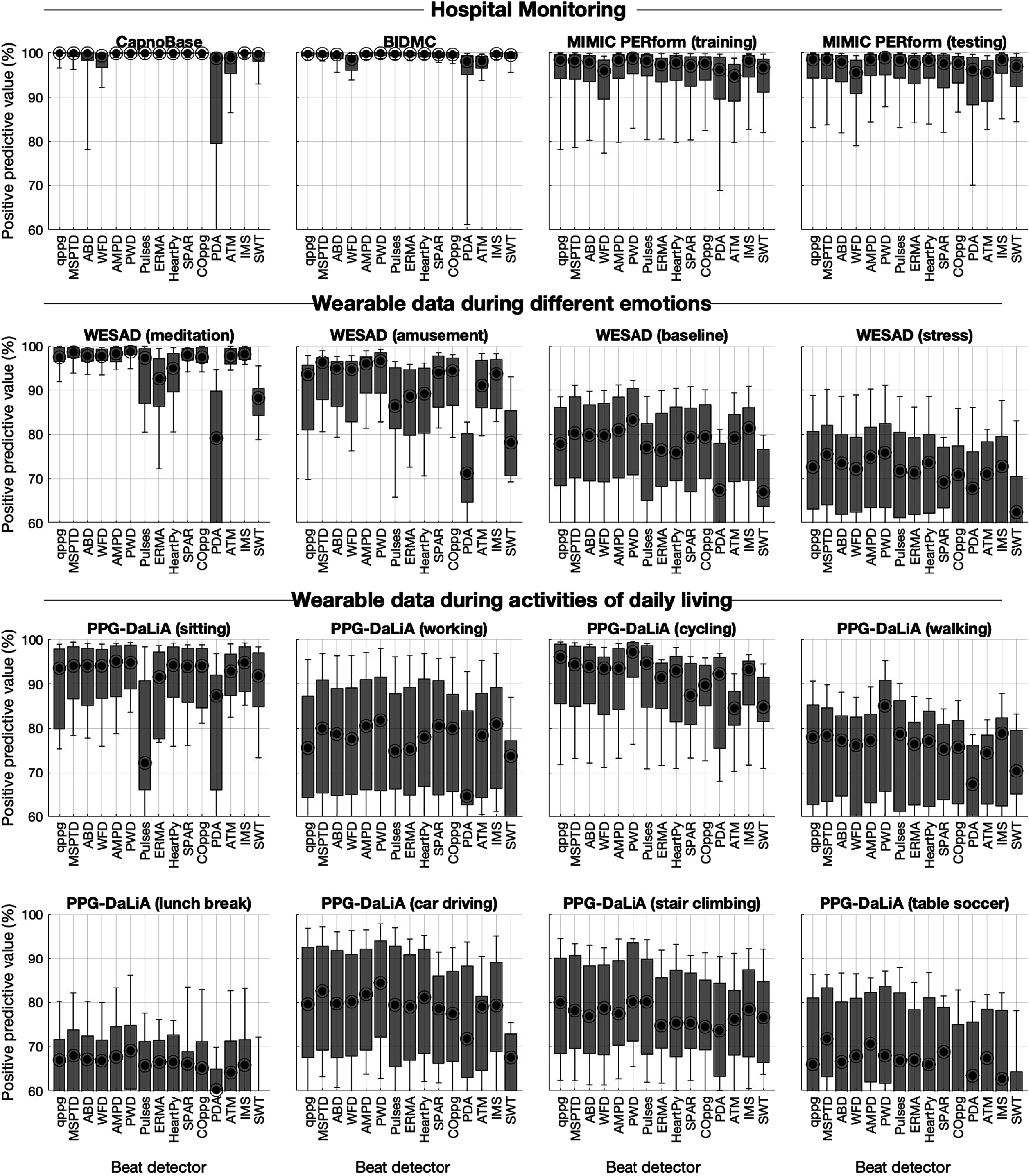
Box plots showing the performance of beat detectors, expressed as the positive predictive value. Each graph shows the results for each of the beat detectors on a particular dataset. Performance is shown as the median (circles), inter-quartile range (boxes), and 10th and 90th percentiles (whiskers) across subjects. See table 2 in the main text for definitions of beat detectors.

The performance of beat detectors is presented in figure 3 using the *F*_1_ score, and in figure 4 using the HR MAPE. Additional results are provided in appendix A for sensitivity and PPV (figures A1 and A2 respectively). The key findings are as follows.

**Figure 5.**
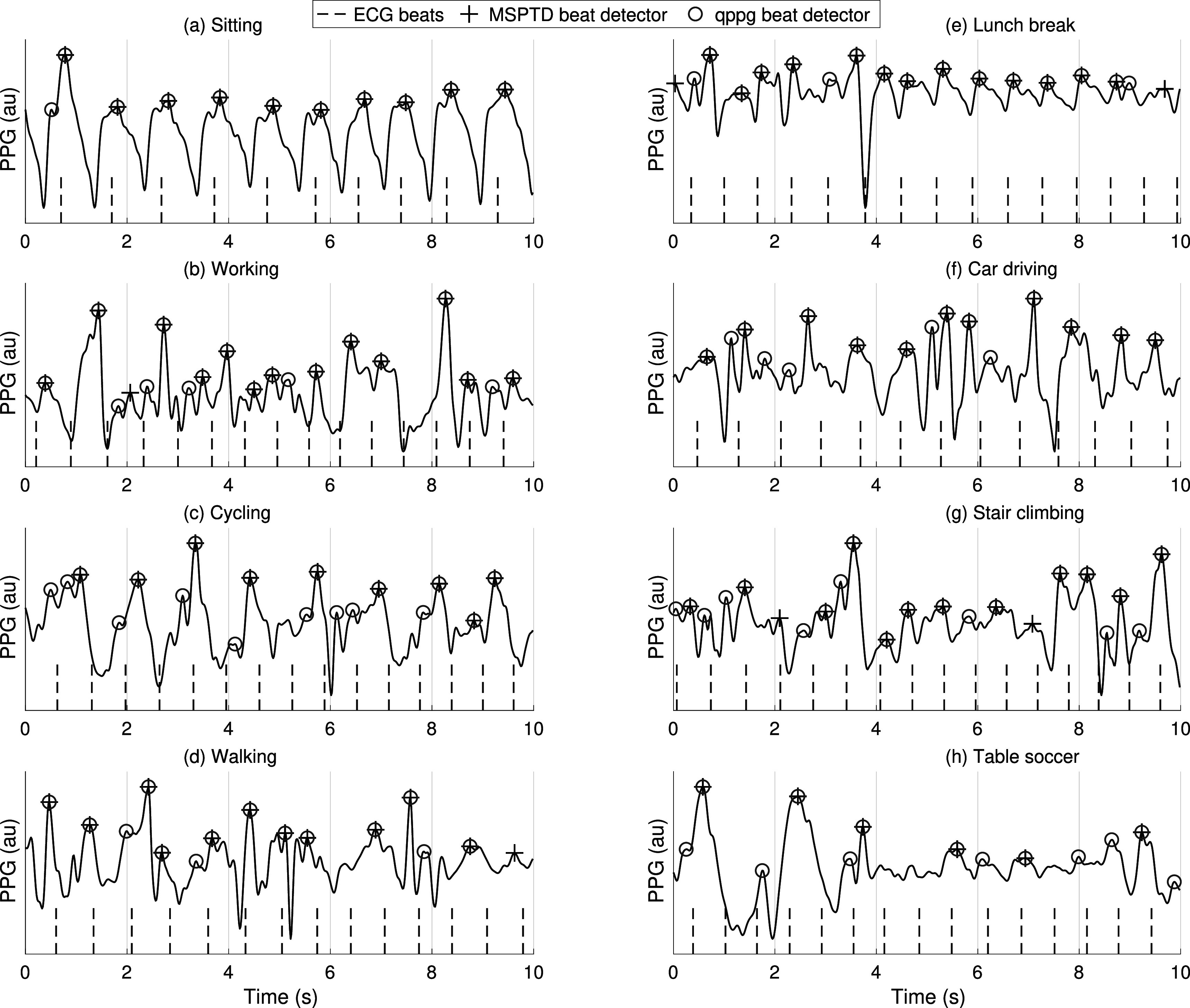
PPG beat detection during different activities: PPG signals are shown for different activities of daily living from the PPG-DaLiA dataset. Beats detected by two PPG beat detectors are shown alongside reference ECG beats. *au—arbitrary units*.

First, eight beat detectors performed very well across all datasets with low levels of movement: AMPD, MSPTD, qppg, PWD, ERMA, SPAR, ABD, and HeartPy. These had median *F*_1_ scores of: ≥99% on the hospital monitoring datasets containing high-quality data (CapnoBase and BIDMC); ≥90% on the hospital monitoring datasets containing real-world data (MIMIC PERform Training and Testing Datasets); and ≥90% on the wearable datasets with low levels of movement (WESAD (meditation) and PPG-DaLiA (sitting)). The remainder of the Results will focus on these eight beat detectors. Figure 5(a) shows an example of (mostly) accurate beat detection during low levels of movement. Of note, the Pulses beat detector performed less well on the PPG-DaLiA (sitting) dataset because its assumed duration of the systolic upslope was no longer valid in these wrist signals acquired at rest.

Second, performance decreased during activities associated with more movement. The eight beat detectors which performed well on data with low levels of movement had median *F*_1_ scores of 93%–96% on PPG-DaLiA (sitting). This performance decreased to 70%–91% on PPG-DaLiA (cycling), 60%–77% on PPG-DaLiA (walking), and 55%–72% on PPG-DaLiA (stair climbing). Performance was also poorer during stress, as shown by median *F*_1_ scores of 59%–70% on WESAD (stress) compared to 71%–80% on WESAD (baseline). This was primarily due to beat detectors missing beats, rather than falsely detecting beats, as shown by the generally lower sensitivities than positive predictive values on PPG-DaLiA (walking) and WESAD (stress) datasets (see appendix A, figures A1 and A2). Figures 5(b)–(d) show examples of beat detection during movement.

Third, the variability in performance between subjects was low during activities associated with low levels of movement, as shown by the relatively low inter-quartile ranges of *F*_1_ scores (indicated by the heights of boxes) on WESAD (meditation) and PPG-DaLiA (sitting). However, performance varied much more between subjects in more challenging datasets, e.g., WESAD (stress) and PPG-DaLiA (walking).

### 3.2. Best-performing beat detectors

To identify the best-performing beat detectors, we focused on results from the MIMIC PERform Testing and PPG-DaLiA (working) datasets, since these are representative of real-world performance in critical care and daily life respectively. On the MIMIC PERform Testing Dataset, the top scoring beat detectors were MSPTD, AMPD, gppq, ABD, and Pulses (all with *F*_1_ scores of 96.6%–97.5%, whereas the remainder scored ≤ 95.6%). On PPG-DaLiA (working), the top scorers were PWD, MPSTD, AMPD, ABD, gppq, and WFD (all with *F*_1_ scores of 80.0%–81.4%, whereas the remainder scored <79.0%). In addition, MSPTD was the best performing beat detector on 5 out of the 12 WESAD and PPG-DaLiA datasets, and qppg was the best performing beat detector on 4 of these datasets. Therefore, we suggest that MSPTD and qppg performed best, although we note that this is subjective, and that some other beat detectors also performed well (notably ABD and AMPD).

The best-performing beat detectors have complementary performance characteristics: MSPTD tended to have a higher positive predictive value, whereas qppg tended to have higher sensitivity (see appendix A, figures A1 and A2). Figure 5 shows examples of this: qppg sometimes detected additional beats during noise (see figure 5(a) at 0.5 s), whereas MSPTD sometimes missed beats (see figure 5(h)).

### 3.3. Acceptability for heart rate monitoring

The performance of beat detectors was deemed to be acceptable for HR monioring in some use cases but not others (see figure 4). All eight beat detectors which had been found to perform well on data with low levels of movement also had acceptable HR MAPEs of <10% on datasets associated with low and moderate levels of movement (the hospital monitoring datasets, and WESAD (meditation, amusement, baseline) and PPG-DaLiA (sitting, working)). At least some of these beat detectors did not perform acceptably on each of the remaining datasets. None of the eight beat detectors produced acceptable HR errors during stress (see WESAD (stress)). Five of the eight beat detectors (MSPTD, qppg, ABD, AMPD, and ERMA) also produced acceptable errors during less intensive activities (PPG-DaLiA (lunch break), and PPG-DaLiA (car driving)). Only qppg performed acceptably on PPG-DaLiA (cycling). None of the beat detectors performed acceptably during more intensive exercise (PPG-DaLiA (walking), PPG-DaLiA (stair climbing), and PPG-DaLiA (table soccer)).

### 3.4. Association between performance and patient physiology and demographics

**Figure 6.**
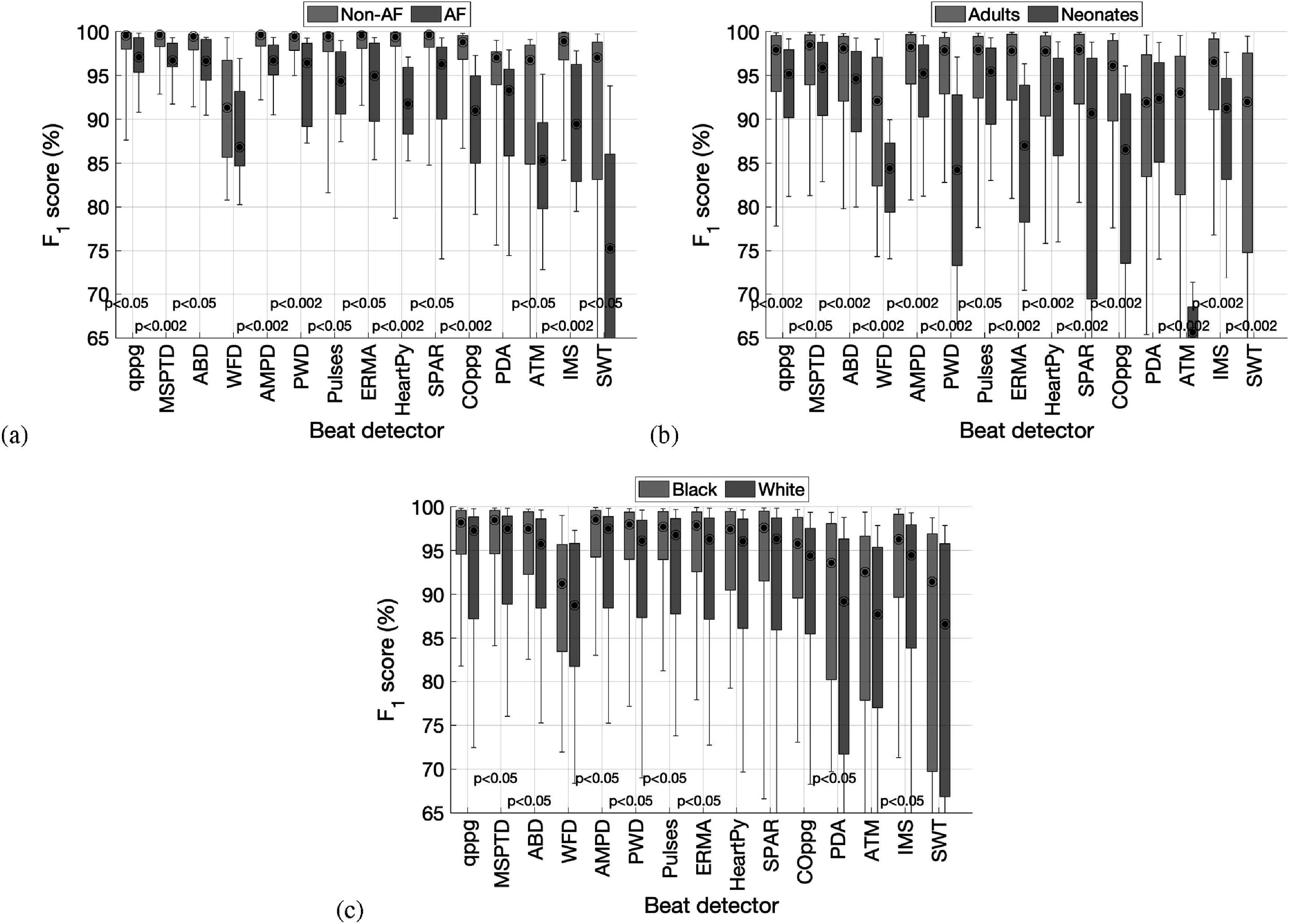
Box plots showing the associations between beat detector performance and patient physiology and demographics. (a) comparison of subjects without and with atrial fibrillation (non-AF and AF); (b) adults compared to neonates; (c) Black compared to White subjects. Performance is shown as the median (circles), inter-quartile range (boxes), and 10th and 90th percentiles (whiskers) across subjects. See table 2 for definitions of beat detectors.

The associations between beat detector performance and the assessed factors are shown in figure 6.

**Figure A3.**
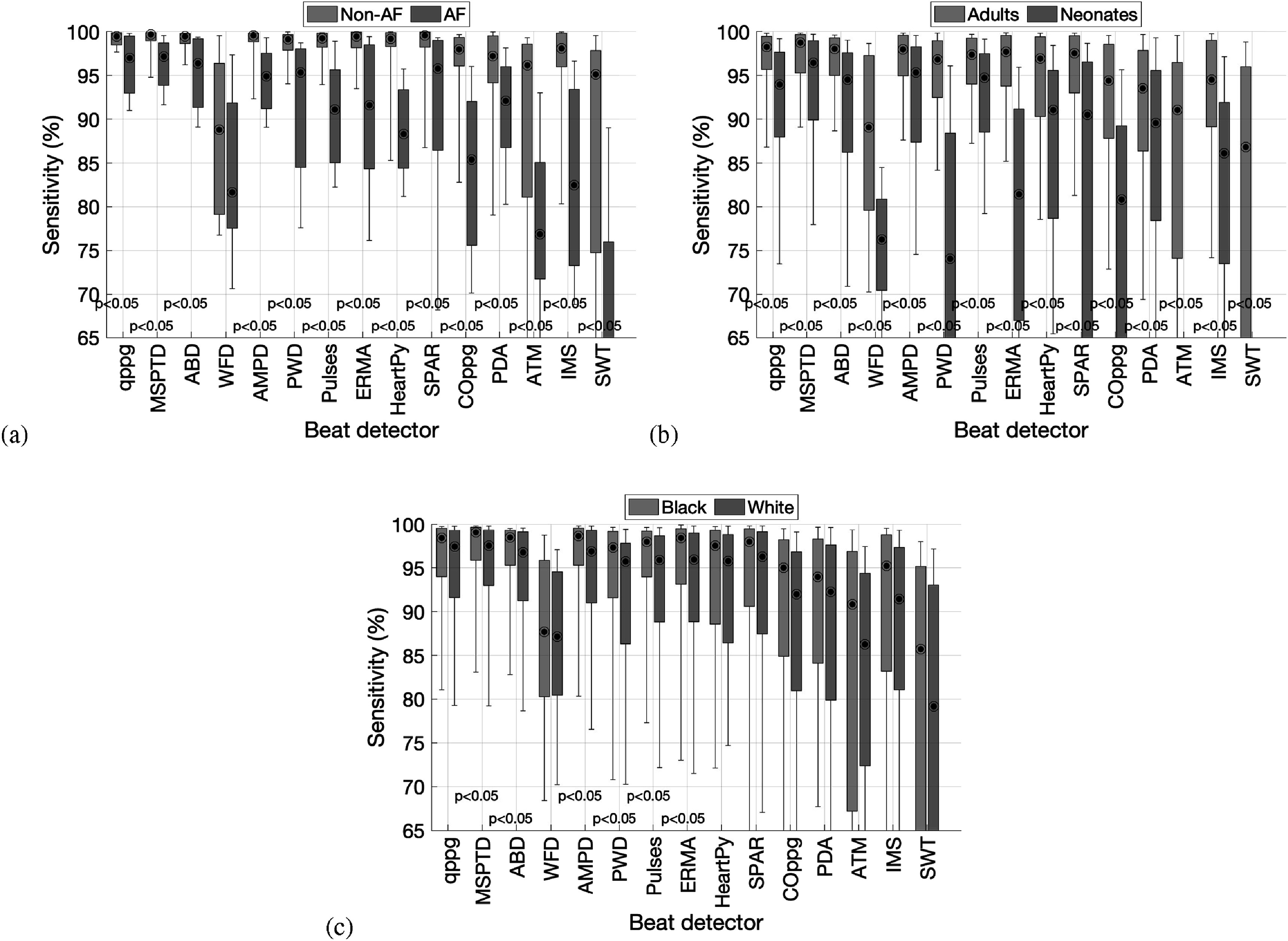
Box plots showing the associations between beat detector performance and patient physiology and demographics, expressed as the sensitivity. Each graph shows the results for each of the beat detectors on a particular dataset. Performance is shown as the median (circles), inter-quartile range (boxes), and 10th and 90th percentiles (whiskers) across subjects. See table 2 in the main text for definitions of beat detectors.

**Figure A4.**
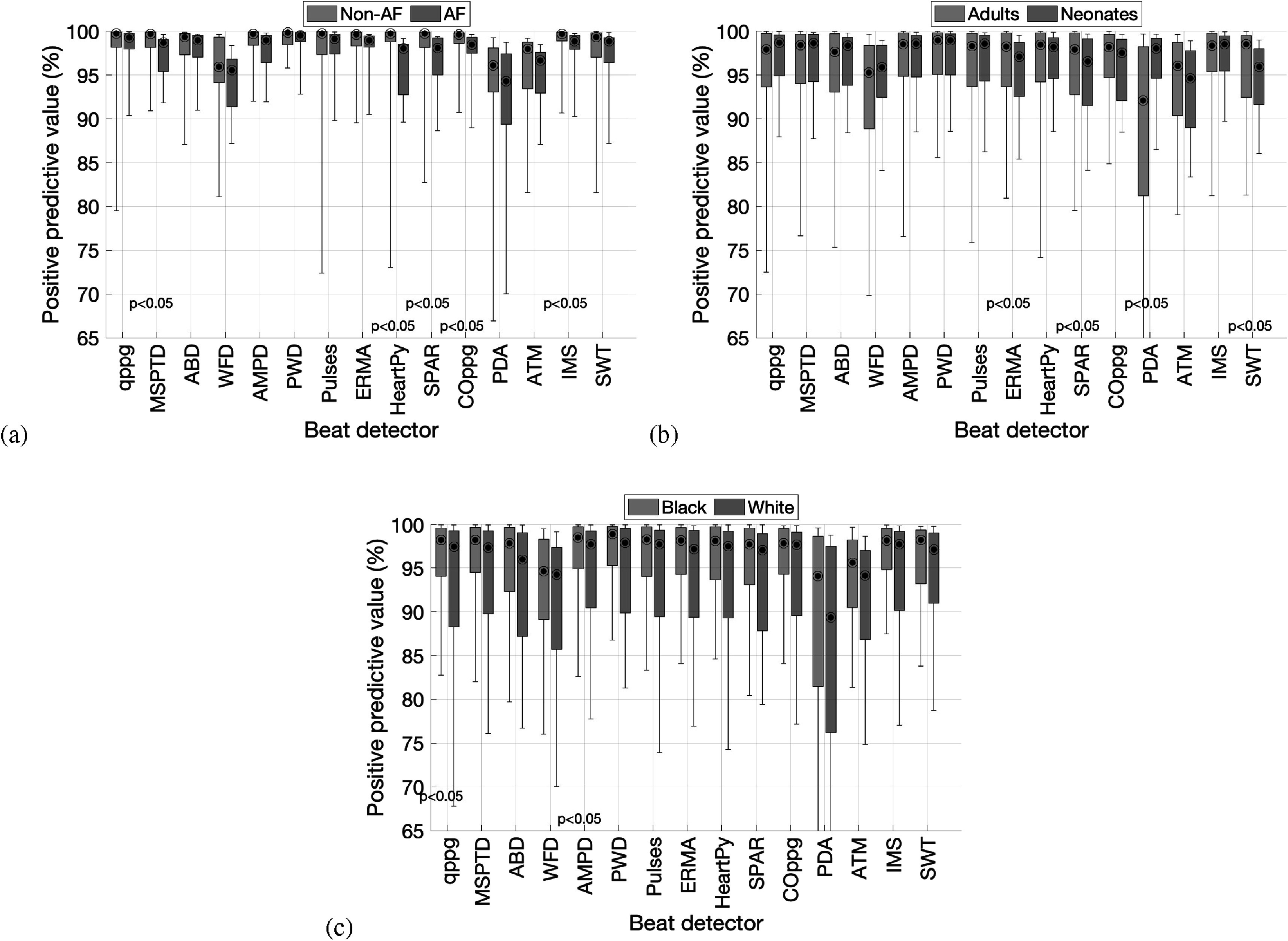
Box plots showing the associations between beat detector performance and patient physiology and demographics, expressed as the positive predictive value. Each graph shows the results for each of the beat detectors on a particular dataset. Performance is shown as the median (circles), inter-quartile range (boxes), and 10th and 90th percentiles (whiskers) across subjects. See table 2 in the main text for definitions of beat detectors.

The performance of beat detectors was poorer in AF (figure 6(a)). The eight beat detectors which performed well at rest achieved *F*_1_ scores between 99.4%–99.7% in sinus rhythm (non-AF), compared to 91.8%–97.1% in AF. This was primarily because beat detectors missed beats during AF (see appendix B, figures A3(a) and A4(a)), similarly to performance in movement. Performance was worse in AF subjects than non-AF subjects for all eight beat detectors at the 5% significance level, and four of these differences remained significant after accounting for multiple comparisons (0.2% significance level).

All eight beat detectors performed worse on neonates than adults, as shown in (figure 6(b)). Seven of these differences remained significant after accounting for multiple comparisons. The eight beat detectors achieved *F*_1_ scores between 97.8%–98.5% in adults compared to 84.2%–95.9% in neonates. These beat detectors missed beats, as shown by their lower sensitivities (see appendix B, figure A3(b)). The lower performance in neonates may be because the neonatal PPG signals were of lower quality, as shown by them having lower SNRs (−10.9 (−12.2 to −8.8) dBc in neonates compared to −5.9 (−9.6 to −1.6) dBc in adults). In addition, some beat detectors may have been designed for use with adults, who typically have HRs between 60 and 100 bpm, whereas neonates typically have HRs between 110 and 160 bpm (Fleming *et al* 2011).

Five of the eight beat detectors had lower *F*_1_ scores on White subjects than Black subjects, as shown in (figure 6(c)), although none of these differences were significant after accounting for multiple comparisons.

### 3.5. Assessment framework

Table 4 presents the proposed assessment framework. The MIMIC PERform datasets are recommended for developing and testing algorithms, and for comparing performance between adults and neonates. Out of the wearable datasets, WESAD is recommended for training and PPG-DaLiA for testing, as the latter allows performance to be assessed during several activities of daily living. The MIMIC PERform AF Dataset is recommended for assessing performance in AF, although it would benefit from inclusion of additional subjects in the future. The CapnoBase and BIDMC datasets were designated as ‘preliminary design’ datasets as all beat detectors achieved *F*_1_ scores of *>*93% on these datasets, so it is unlikely they could be used to substantially improve beat detector design.

**Table 4.** The proposed assessment framework.

Purpose	Dataset	Data access
*Algorithm Development*		

Preliminary design	CapnoBase	Available in Matlab format after completing an agreement.
Preliminary design	BIDMC	Available in CSV, WaveForm DataBase, and Matlab format, under an OCD-By licence.
Design with critical care data, and compare performance in adults and neonates	MIMIC PERform Training Dataset	Available in Matlab, CSV and WaveForm DataBase formats, under an ODb licence.
Design with wearable data	WESAD	Available in Python’s PKL format, for non-commercial purposes.
Investigate impact of atrial fibrillation	MIMIC PERform AF Dataset	Available in Matlab, CSV and WaveForm DataBase formats, under an ODb licence.

*Algorithm Testing*		

Testing with critical care data	MIMIC PERform Testing Dataset	Available in Matlab, CSV and WaveForm DataBase formats, under an ODb licence.
Testing in activities of daily living	PPG-DaLiA	Available in Python’s PKL format, for non-commercial purposes.

## 4. Discussion

This study assessed the performance of several open-source PPG beat detectors across a range of datasets. Most beat detectors performed well on hospital data and at rest, but performed worse during movement, stress, AF, and in neonates. The study provides a standardised framework with which to develop and test beat detectors.

The findings could inform PPG-based monitoring strategies and directions for algorithm development. The poorer performance of beat detectors during movement is reflected in current monitoring strategies. For instance, smartwatches which use the PPG to check for an irregular pulse often only do so whilst the subject is stationary (Perez *et al* 2019) - a strategy which is supported by this study. Future work should investigate how best to use a simultaneous accelerometry signal to identify periods in which the subject is stationary and therefore beats can be accurately detected. The poorer performance in neonates and during AF indicates areas for development (Han *et al* 2022). Future work could also assess performance in other situations which impact the pulse wave, such as during ectopic beats, hypoperfusion, and vascular disease. This study also provides motivation for strategies to improve beat detection and exclude unreliable data from analyses, such as motion artifact cancellation and signal quality assessment.

The beat detectors used in this study are indicative of the range of approaches proposed in the literature to detect beats in the PPG. As detailed in table 2, approaches included: (i) identifying peaks in the original PPG signal (HeartPy and COppg); (ii) identifying systolic upslopes using the original signal (IMS) or first derivative (qppg, ABD, PWD and Pulses); (iii) using the local maxima scalogram to identify peaks across several scales (MSPTD and AMPD); and (iv) representing the PPG in phase space (SPAR). The MSPTD and qppg beat detectors performed best in this study. MSPTD searches for peaks without using any prior knowledge of the characteristics of PPG pulse waves. In contrast, qppg searches for systolic upslopes based on their expected characteristics. In the future, different approaches could be combined to improve performance.

The algorithms, datasets, and assessment framework used in this study are all freely available. This has several benefits. Firstly, it ensures that the study is reproducible. Secondly, it allows others to assess the performance of their own beat detection or quality assessment algorithms. Thirdly, the framework provides a basis with which to design (using the training datasets) and test such algorithms. Since the training datasets contain a variety of challenges, such as different use cases and populations, we expect that developers will benefit from using this framework for algorithm development. The framework cannot be considered to be exhaustive, and datasets recorded in additional settings and from further patient populations, could be added in the future. These resources and corresponding documentation are archived at Charlton (2022a, 2022b), whilst the most up to date version can be obtained at: https://github.com/peterhcharlton/ppg-beats.

The key limitations are as follows. First, the study is limited to open-source beat detectors, rather than all those reported in the literature (see (Charlton *et al* 2022) for a description of additional beat detectors). Second, no attempt was made to improve the algorithms, but rather this study established the performance of existing algorithms. Third, some datasets were relatively small: WESAD and PPG-DaLiA contain data from 15 subjects, and the MIMIC PERform AF Dataset contains data from 35 patients. Fourth, the framework assumes that pulse arrival time (PAT) is constant within a subject’s recording, which is reasonable for the short recordings in this study, but changes in PAT should be accounted for if using longer recordings (Kotzen *et al* 2021).

## 5. Conclusions

This study demonstrated the high performance of the MSPTD and qppg beat detectors across a range of use cases. Most beat detectors performed well in the absence of movement, whereas performance was poorer during stress, activities of daily living, in neonates, and during AF. The results inform key directions for future work: (i) improving performance in neonates and during AF; (ii) investigating whether motion artifact cancellation improves performance; and (iii) investigating whether algorithms to assess signal quality can distinguish between periods in which beats can or cannot be accurately detected. The algorithms, datasets, and assessment framework used in this study are all publicly available in Charlton (2022a, 2022b).

## Data Availability Statement

The data that support the findings of this study are openly available at the following URL/DOI:10.5281/zenodo.6807402.

## Competing interests

P. J. Aston has a patent (WO2015121679A1 ‘Delay coordinate analysis of periodic data’), which covers the foundations of the SPAR method used in this paper.

## Appendix A. Performance of PPG beat detectors in different use cases

The performance of photoplethysmogram (PPG) beat detectors in different use cases was presented in figure 3 in the main text, using the *F*_1_ score to describe performance. Additional results are shown in: figure A1, which shows the sensitivity of beat detectors; and figure A2, which shows their positive predictive value.

## Appendix B. Association between PPG beat detector performance and patient demographics and physiology

Associations between PPG beat detector performance and patient demographics and physiology were presented in figure 5 in the main text, using the *F*_1_ score to describe performance. Additional results are shown in: figure A3, which shows the sensitivity of beat detectors; and figure A4, which shows their positive predictive value.
